# When ART fails: figures, experiences, interventions and a plea for the ‘plan B’

**DOI:** 10.1007/s10815-021-02388-7

**Published:** 2022-01-04

**Authors:** Tewes Wischmann, Petra Thorn

**Affiliations:** 1 Institute of Medical Psychology, Heidelberg University Hospital, Bergheimer Str. 20, D-69115 Heidelberg, Germany; 2 Practice for Family Therapy and Infertility Counselling, Moerfelden, Germany

**Keywords:** Success rates, Childlessness, Plan B, Counselling

## Abstract

Infertility is perceived by many of those affected by it as one of the most stressful episodes in life. Assisted reproduction can help only some of the people with a desire for children to experience the birth of a biological child. Most people who remain involuntarily childless eventually come to terms with the situation; their psychological well-being is not lastingly affected. However, they should envisage a ‘plan B’ as early as possible. The prospect of permanent childlessness should not be an unmentionable topic, neither for couples themselves nor for the doctors treating them.

‘Failures (with some successes) of assisted reproduction and gamete donation programs’ [1] is the (slightly provocative) title of an article in the *Human Reproduction* journal which—for once—focuses on those instances where reproductive medicine fails to ‘deliver the goods’. The present article is an opinion paper that begins with critical observations on the prospects of success with assisted reproduction. This is followed by an account of the outcomes of a study on the quality of life of involuntarily childless people. Finally, we outline some examples of interventions undertaken in the context of psychosocial infertility counselling and indicate potential helpful responses physicians can draw upon in the event of assisted reproductive treatment (ART) ultimately failing.

For most people wanting a child, the most interesting figure associated with reproductive medicine is the live birth rate and transparent and customized information about the chance of having a baby, considering the couple’s circumstances (e.g. age, medical diagnoses). For couples using their own gametes, however, this is only approximately 20% per treatment cycle [2]. And if we regard not *completed* ART cycles but cycles that have been *embarked upon*, then the figures are even less encouraging. Of 12 couples embarking on an IVF or ICSI treatment cycle, ten will make it to embryo transfer, three of the women will get pregnant, and two couples will take a baby home [3]. These figures are the averages of all ART treatments for women of all ages, all medical diagnoses and all treatment centres in Germany. Accordingly, the outcomes in individual cases may be substantially more favourable—or considerably less so. Taken in conjunction with the cumulative birth rate after ART, the figures are a fairly reliable approximation for psychosocial infertility counselling to work with. As such, they indicate very clearly the necessity of envisaging a ‘plan B’. The unsuccessfulness of ART—no live birth of a child—therefore is a likely option [4].

## Miscarriages and live births after ART

In the discussion on pregnancies after ART, miscarriages are frequently given insufficient attention. Per treatment cycle, the miscarriage rate is approximately 20% after IVF/ICSI fresh cycles and 25% in the case of cryotransfer, i.e. much higher than with spontaneous conception [5]. The same applies to the risk of extra-uterine pregnancy and stillbirth after ART. Most couples undergoing ART perceive miscarriages as if ‘under a microscope’. Women receiving infertility treatment are normally subjected to close-meshed medical ultrasound monitoring that makes it almost impossible to overlook even a very early miscarriage [6].

**Fig. 1:**
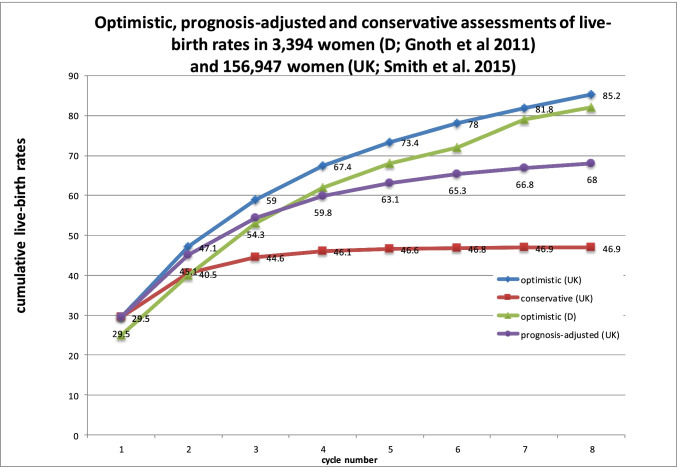
Cumulative live birth rates

How many people actually crown the assisted reproduction process with the live birth of a child? Fig. 1 shows the respective cumulative birth rates recorded by two studies, one British [7] and the other German [8].

Up to half of the couples are ‘dropouts’, i.e. they cease ART before a live birth and there is no record of the further course of their lives [9]. Accordingly, assessments are necessary to obtain an approximate idea of the actual success rates of infertility treatment. The optimistic assessment (green and blue lines in Fig. 1) proceeds on the assumption that all dropouts have the same pregnancy prospects as the couples who have persevered with treatment. By contrast, the conservative assessment assumes that the dropouts will not achieve pregnancy in the future either (red line in Fig. 1). It is thus fair to assume that the ‘true’ scores will lie somewhere between the two percentages. Accordingly, the ‘prognosis-adjusted’ assessment (mauve line in Fig. 1) assumes that approximately 30% of the dropouts will no longer achieve pregnancy. In both the studies referred to here, about half the couples on average take a baby home with them after three ART cycles (baby-take-home rate) and about 60% of the couples after four attempts. Up to the sixth cycle, the cumulative live birth rate increases to approx. 66% on average; further attempts make little sense statistically.

A recent study (based on the SART data for the USA over the years 2014–2016) including 88,613 women who commenced IVF treatment using their own eggs and partner’s sperm came to similar results: ‘Overall, 55.9% of all couples had a live birth over the first three complete cycles of IVF during the 3-year study period’ [10].

A French study retrospectively explored the achievement of parenthood 8 years after starting IVF of 6,507 couples [11]. Forty-eight percent live births were counted after ART, 12% live births after spontaneous conception and 11% adoptions. This means that the cumulative live birth rate after ART is in fact only about 50%.

## ART and dropout

According to the ESHRE guideline, approximately 25–30% of women/couples (range: 17–70%) cease ART without the live birth of a child [12]. The main reasons for this are poor prospects of success (either from the subjective perspective of the women/couples or on the basis of medical assessment) and psychological stress.

## Emotional repercussions after an unsuccessful ART treatment cycle

According to the ESHRE guideline, the effects of an unsuccessful ART cycle may be serious. After receiving a negative test outcome following IVF/ICSI treatment, 1 out of 4 women and 1 out of 10 men display a depressive disorder, while 1 out of 7 women and 1 out of 20 men suffer from an anxiety disorder. Patients with low acceptance of their sub-fertility/childlessness and the experience of marked helplessness in the face of their diagnosis and the infertility treatment display more pronounced degrees of anxiety and depression after unsuccessful treatment [12].

## Medium- and long-term repercussions of lasting infertility

Two years after unsuccessful infertility treatment, the ESHRE guideline [12] indicates that on average most couples are generally content with their partnership. After 5 years, by contrast, a Swedish study [13] finds them to be more vulnerable than parents who have undergone infertility treatment to indulgence in hedonic items (bad-for-you’ s) and the use of sleeping pills, and the separation rate is also three times as high as in the comparison group. However, an extensive follow-up study 10 to 14 years after the termination of treatment [14] comes to different conclusions. Only 17% of the women reported that they had separated from the partner they had gone through infertility treatment with (separation rates in the overall population are 25–30%).

## Recommendations for infertility counsellors

One of the central interventions in infertility counselling is the suggestion to draw up ‘roadmaps’ [15]. Couples are encouraged to take those things into their own hands that they can readily influence: the tempo of infertility therapy and its degree of invasiveness (e.g. from hormone stimulation to inseminations, from inseminations to ART), decisions on breaks and above all on limits. The partners are asked to come up with roadmaps for all the various eventualities (no pregnancy, miscarriage, childbirth) and to include ‘plan B’ and ‘plan C’ from the outset. Initially, each individual partner can elaborate a roadmap of their own; subsequently the couples compare their maps and dovetail them with one another. If so desired, the roadmaps can also be rewritten at a later stage (e.g. after the first unsuccessful IVF attempt), but the couple should *always* impose limits [‘healthy limits’, 16] on infertility treatment [15].

In counselling, one recommendable way of convincing the couple of the importance of working out ‘plan B’ at an early stage is the following: if after (possibly) a number of years, the treatment with reproductive medicine should turn out to be unsuccessful and the couple had to fall back on this plan B, then they would already have it ‘in the drawer’. And if the infertility treatment was ultimately successful, then that’s where it could stay [4, p. 69]. One good example of a ‘plan B’ was posted in an internet infertility forum: ‘We also have a plan B. Every week from now on, we’ll be writing down things we enjoy (e.g. a particular meal), things we like doing (e.g. going on town trips) and things we’re planning to do later (e.g. a safari) and putting them in a receptacle. All those bits of paper will accumulate over time and keep us aware of the good things in our lives, the things we can be glad about right now and the things we can look forward to later on.’ [17].

Counselling experts should be unequivocal in pointing out that the differences in life quality between non-parents and parents are slight. Favourable in prognostic terms are the positive re-evaluation and acceptance of the situation plus the active search for alternatives and social contacts. Prospectively unfavourable features are brooding and avoidant coping, feelings of powerlessness and adherence to a single-minded focus on children as a life-aim. One of the most potent risk factors militating against success in coming to terms with involuntary childlessness is social isolation [18].

The couple should be prepared for the fact that the entire process may leave a ‘scar’ that can break open again in later life when friends become grandparents. The desire for a child is one of the most cherished dreams in the life of the couple as a couple. If that dream fails to materialize, the loss can never be healed but only mitigated, and the cracks will always be visible (much as in Japanese *kintsugi*, where new artworks evolve from broken porcelain through explicit foregrounding of the mends).

Finally, it is highly apposite in counselling to point out that ultimately the effort involved in coming to terms with grief will release those energies formerly held in check by the vicissitudes of medical infertility treatment [4]. These are the good news along with the bad news [19].

## Desiderata: recommendations for physicians

Reproductive medicine often develops a momentum of its own that can take on the character of addiction. Accordingly, the need for a ‘plan B’ should be addressed right at the beginning of the infertility journey [15, 20]. Here is an illustrating comment made by a woman after undergoing infertility therapy without success: ‘Medical care could be improved by avoiding the excessively cautious wording in which “bad” results are often couched. For me, this was a recurring source of deceptive hope, relegating the eventuality of never having children to the background. And when we finally had to face up to that brute fact, it was a very hard blow to take. In my view, the likelihood of things not working out well should have been referred to more regularly during the treatment process.’ [from 3, p. 141]. While emotionally the prospect of permanent childlessness is indeed challenging in the extreme, the eventuality should not become an unmentionable topic for the couple involved [21].

If a cycle is unsuccessful, the strategy of choice is to emphasize the failure of therapy, not any personal responsibility on the part of the person/couple [22]. Having a ‘plan B’ to fall back on will certainly not reduce the likelihood of pregnancy, just as ‘positive thinking’ alone is no sure-fire prerequisite for getting (and staying) pregnant [4]. True, pronounced pessimism can prevent couples from going through all the treatment cycles even if the individual prospects for successful ART are positive. But optimism is by sure not an indispensable precondition for embarking on such treatment: this is clearly a myth. This is the central message of this opinion paper. Accordingly, at the very first interview at the infertility treatment centre, the physician in charge should openly and squarely address the necessity of having a ‘plan B’ to resort to: ‘We will do everything in our power to help you fulfil your dream of a biological child. But what will you do if we are unsuccessful?’ While the prospect of permanent childlessness is certainly challenging in the extreme, it should not become an unmentionable topic, *either* for the couple *or* for the doctors.

## Proposals for future research (e.g. [18])

High-quality prospective studies on the effects of unsuccessful infertility treatment should be planned and carried out for all groups of patients (heterosexual and lesbian couples, single mothers by choice). Furthermore, presenting response rates should be a standard feature in follow-up studies (with accurate responder/non-responder analyses). As it is fair to assume that women and men with different ethnic, religious, societal, and cultural backgrounds will also differ in their evaluation of the significance of parenthood, these moderator variables should be included in studies on permanent unintentional childlessness. Finally, preventive interventions mitigating the repercussions of permanent infertility need to be developed and evaluated, as well as the evaluation of psychosocial interventions for persons with fertility problems.

## Declarations

### Disclaimer

The authors alone are responsible for the content and writing of the paper.
